# The impact of C-reactive protein levels on headache frequency in the HUNT study 2006–2008

**DOI:** 10.1186/s12883-019-1462-8

**Published:** 2019-09-26

**Authors:** Knut Hagen, Lars Jacob Stovner, Kristian Bernhard Nilsen, Espen Saxhaug Kristoffersen, Bendik Slagsvold Winsvold

**Affiliations:** 1 Department of Neuromedicine and Movement science, NTNU Norwegian University of Science and Technology, 7491 Trondheim, Norway; 2 Norwegian Advisory Unit on Headache, St. Olavs Hospital, Trondheim, Norway; 3 Clinical Trial Unit, St. Olavs Hospital, Trondheim, Norway; 4 Department of Neurology, Oslo University Hospital, Oslo, Norway; 5 Department of General Practice, HELSAM, University of Oslo, Oslo, Norway; 6 Department of Neurology, Akershus University Hospital, Lørenskog, Norway; 7 Department of Neurology and FORMI, Oslo University Hospital, Oslo, Norway; 8 Institute of Clinical Medicine, University of Oslo, Oslo, Norway

## Abstract

**Background:**

Increased high sensitivity C- reactive protein (hs-CRP) levels have been found in many earlier studies on migraine, and recently also in persons with migraine and insomnia. The aim of this study was to see whether these findings could be reproduced in a large-scale population-based study.

**Methods:**

A total of 50,807 (54%) out of 94,194 invited aged ≥20 years or older participated in the third wave of the Nord-Trøndelag Health Study study performed in 2006–2008. Among these, 38,807 (41%) had valid measures of hs-CRP and answered questions on headache and insomnia. Elevated hs-CRP was defined as > 3.0 mg/L. The cross-sectional association with headache was estimated by multivariate analyses using multiple logistic regression. The precision of the odds ratio (OR) was assessed with 95% confidence interval (CI).

**Results:**

In the fully adjusted model, elevated hs-CRP was associated with migraine (OR 1.14, 95% CI 1.04–1.25) and migraine with aura (OR 1.15, 95% CI 1.03–1.29). The association was strongest among individuals with headache ≥15 days/month for any headache (OR 1.26, 95% CI 1.08–1.48), migraine (OR 1.62, 95% CI 1.21–2.17), and migraine with aura (OR 1.84, 95% CI 1.27–2.67). No clear relationship was found between elevated hs-CRP and headache less than 7 days/month or with insomnia.

**Conclusions:**

Cross-sectional data from this large-scale population-based study showed that elevated hs-CRP was associated with headache ≥7 days/month, especially evident for migraine with aura.

## Background

During the last decade many studies have evaluated the association between migraine and high sensitivity C-reactive protein (hs-CRP) [1–25]. Several case-control studies [3, 7, 8, 14, 18–22] and some population-based studies [4, 6] have found higher CRP values in migraine patients, but negative results have been reported by others [2, 5, 6, 10, 11, 15, 16, 24]. A summary of recently published studies concluded that migraineurs had higher CRP levels than controls [26].

The association between headache and insomnia is well-established [27], but the impact of insomnia on the relationship between hs-CRP and migraine is unclear. Previously, a prospective study has demonstrated higher level of CRP in individuals with shorter sleep duration [28]. In a recent population-based cross-sectional study from Northern Norway, elevated hs-CRP was associated with migraine, but only among those with co-existing insomnia [29]. However, since the results on hs-CRP and migraine are conflicting, and the relationship to insomnia only reported in one study, it is important to replicate the analyses in other population-based study with similar design.

The aim of this large-scale population-based study was to evaluate the cross-sectional association between hs-CRP and types of headache, and to see whether the relationship to insomnia could be reproduced.

## Methods

### Study design

The present population-based study of a large unselected adult population replicate the methodological design used in the Tromsø Study [29], including similar questionnaire-based diagnoses for headache and insomnia, identical categories of hs-CRP, and replication of the statistical analyses with adjustments for the same type of potential confounders.

### The Nord-Trøndelag health study

The total population of the Nord-Trøndelag county aged ≥20 years has been invited four times between 1984 and 2019. All participant answered questionnaires and were invited to interview, clinical examination, and blood sampling [30].

### Study population

**Fig. 1:**
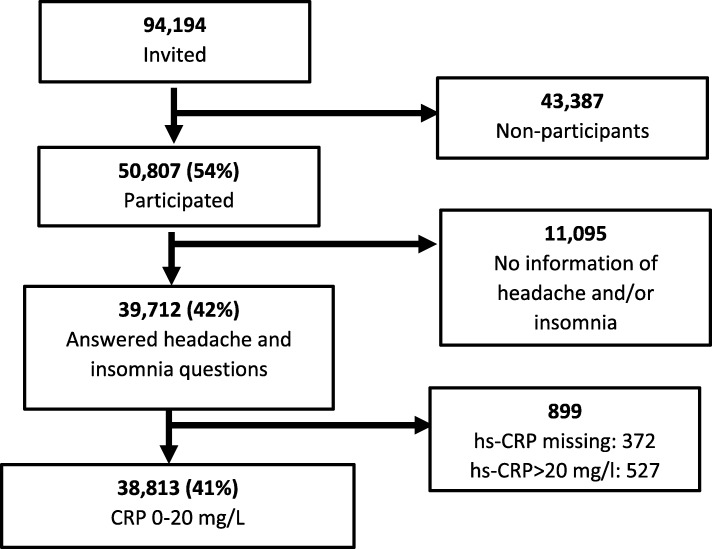
Flowchart of participation in the HUNT Study 2006–2008. Hs-CRP = high sensitivity C-reactive protein

In HUNT3 (2006–2008), a total of 94,194 individuals aged ≥20 years were invited, whereof 93,860 were eligible. Of these, 50,807 (54%) accepted the invitation. The diagnoses of headache and insomnia was questionnaire-based. The present study included data from 38,813 participants (41%) (mean age 54.1 years, range 20–97 years) who had answered questions about headache and insomnia and had hs-CRP measured from non-fasting blood samples (Fig. 1).

### C-reactive protein

Hs-CRP was analyzed at the Central Laboratory, Levanger Hospital, using Architect cSystem ci8200, by latex immunoassay method. The detection limit was 0.03 mg/L, and samples without detectable hs-CRP were assigned this value. In the present study we replicated the strategy used in previous published studies from the Tromsø study [29, 31] defining normal hs-CRP as 0–3.00 mg/L and elevated as 3.01–20.00 mg/L. Participants with hs-CRP values > 20.00 mg/L, which probably indicate some acute or chronic disease [32], were excluded (*n* = 527) (Fig.1). Blood samples for hs-CRP analyzes were drawn without any knowledge about last headache attack.

### Headache questionnaire

All details of the headache questionnaire have been published elsewhere [33], and all questionnaire-based headache diagnoses were identical to those presented in the Tromsø study [29]. Subjects who answered “no” to the screening question (“Have you suffered from headache during the last 12 months?”) were included in the reference group without headache.

Participants who answered “yes” completed 12 additional questions, mainly to diagnose migraine according to the second version of the International Classification of Headache Disorders (ICHD-2) [34]. Regarding attack duration, < 4 h were accepted for those who reported visual disturbance before headache. Participants who fulfilled the migraine diagnosis and reported visual disturbance prior to headache were classified as migraine with aura (MA), whereas the remaining with migraine had migraine without aura (MO). The remaining participants with headache were classified as having “other headache”. The merged group of “any headache” consisted of participants with migraine or other headache. Furthermore, based to answer on the question about headache frequency, subjects were subdivided into five groups; no headache, headache < 1 days/month, headache 1–6 days/month, headache 7–14 days/month, or headache ≥15 days/month.

The validity of the questionnaire-based headache diagnoses has been published in a separate paper [39]. The agreement between the validation interview and questionnaire-based headache categories were very good for any headache (kappa value at 0.70, 95% CI 0.61–0.79) and moderate for migraine (kappa value 0.50, 95% CI 0.32–0.68), whereas the specificity for MA was high (95%) (kappa value 0.44, 95% CI 0.38–0.50) (33).

### Questionnaire-based diagnosis of insomnia

The questionnaire-based diagnosis of insomnia was based on the DSM-V (Diagnostic and Statistical Manual of Mental Disorders, 5th ed.), and was nearly identical to the insomnia diagnosis used in the Tromsø study [29]. To fulfil the proxy diagnosis of insomnia the participants had to answer “several times a week” to at least one out of the three questions, asking whether they had “difficulties falling asleep at night”, whether they “woke up repeatedly during the night”, and/or whether they “woke up too early and could not get back to sleep” [36]. In addition, they also had to report “several times a week” to the question “felt sleepy during day” [36]. The overall agreement between questionnaire and interview for the first three questions has previously been found to be moderate (kappa value 0.51, 95% CI 0.40–0.63) [37].

### Potential confounders

The selection of potential confounders used in the Tromsø study [29] was based on previous literature [35, 37]. To replicate the Tromsø study [29], we included almost identical potential confounders, with two exceptions: employment status was used instead of education level, and Hospital Anxiety and Depression Scale instead of Hopkins symptom checklist (HSCL-10). The remaining potential confounders were identical; age (continuous variable); gender; body mass index (BMI) (< 25, 25.0–29.9, and ≥ 30 kg/m^2^) (28, 32); smoking (current, previous, and never); physical activity (never, ≤ 1 time per week, ≥2 times per week); alcohol consumption (never, < 2 times/week, ≥2times/week); self-reported diabetes (yes/no); self-reported stroke and/or heart infarction (yes/no); and self-reported hypertension (yes/no).

### Ethics

This study was approved by the Regional Committee for Medical and Health Research Ethics, the Faculty of medicine, mailbox 8905, 7491 Trondheim. The approval number was #2018/2422/Rek Midt. The participants have given written informed consent.

### Statistical analysis

The statistical method was identical to that presented in the Tromsø Study [29] and based on multivariate analyses using multiple logistic regression. The precision of the odds ratio (OR) was assessed with 95% confidence interval (CI). We present results for three different statistical models separated by number of confounders included; model 1 (age and sex), model 2 (age, sex and BMI), and model 3 (all other factors were tested). In model 3 we excluded factors if they did not change OR when evaluating each factor separately or when including several factors grouped together (i.e. self-reported diabetes, stroke/heart infarction, and hypertension). Potential interaction between two variables was evaluated by including the product of the variable in the logistic regression analyses, and the interaction was tested using Wald χ^2^ statistics. To evaluate the probability of a linear relationship between elevated hs-CRP and headache frequency, the five categories were treated as a single variable and was incorporated in a two-sided test for trend. Subjects with missing data of confounding factors (numbers reported in Table 1) were included in the analysis to reduce the impact of possible bias.

**Table 1:** Characteristics of participants (*n* = 38,813) in the HUNT3 study related to headache types

	No headache	Any headache	MA	MO	Other headaches
Participants, n	24,823	13,990	2139	1654	10,197
Women, n (%)	12,525 (50.5)	9203 (65.8)	1600 (74.8)	1252 (75.7)	6351 (62.2)
Mean age, years (SD)	56.9 (15.7)	49.2 (14.2)	47.0 (12.9)	45.3 (12.6)	50.2 (14.6)
Age ≥ 60 years (%)	11,280 (45.4)	3264 (23.3)	358 (16.7)	219 (13.2)	2687 (26.4)
Employed, n (%) (missing = 17)	14,450 (58.2)	10,087 (72.1)	1570 (73.4)	1301 (78.7)	7216 (70.8)
Current daily smoking, n (%) (missing = 998)	3590 (14.5)	2534 (18.1)	497 (23.2)	277 (16.7)	1760 (17.3)
Mean BMI, kg/m^2^ (SD) (missing = 120)	27.2 (4.2)	27.2 (4.7)	27.3 (4.8)	27.1 (5.0)	27.2 (4.6)
Total HADS score (missing = 582)	6.7 (5.0)	8.3 (5.9)	9.4 (6.4)	8.2 (5.9)	8.1 (5.8)
Alcohol abstainers during last year, n (%) (missing = 956)	2215 (8.9)	1105 (7.9)	120 (7.3)	182 (8.5)	803 (7.9)
Physical inactivity, n (%) (missing = 615)	1231 (5.0)	574 (4.1)	69 (3.2)	63 (3.8)	442 (4.3)
Self-reported hypertension, n (%)	6503 (26.2)	2462 (17.6)	359 (16.8)	215 (13.0)	1888 (18.5)
Self-reported stroke and/or heart infarction (%)	1771 (7.1)	506 (3.6)	63 (2.9)	33 (2.0)	410 (4.0)
Self-reported diabetes mellitus, n (%)	1326 (5.3)	466 (3.3)	60 (2.8)	34 (2.1)	372 (3.6)
Modified DSM-V insomnia, n (%)	1057 (4.3)	1631 (11.7)	362 (16.9)	235 (14.2)	1034 (10.1)
Mean hs-CRP (95% CI)	2.2 (2.2–2.3)	2.3 (2.2–2.3)	2.4 (2.3–2.5)	2.2 (2.1–2.4)	2.3 (2.2–2.3)

*MA* Migraine with aura, *MO* Migraine without aura, *HADS* Hospital anxiety and depression scale, *BMI* Body mass index, *hs-CRP* High sensitivity C-reactive protein, *CI* Confidence intervals, *DSM-V* Diagnostic and statistical manual of mental disorders, 5th ed.

To evaluate insomnia as a modifying factor on the association between hs-CRP and types of headache, we repeated the multi-adjusted analyses in model 3 in those with and without insomnia.

Analyses were performed with the IBM SPSS version 25 (SPSS, Chicago, Illinois, USA).

## Results

Subjects with headache were more likely to be women, younger, and to be employed in paid work compared to those without headache (Table 1).

### Prevalence of headache

A total of 13,990 (36.0%) participants suffered from headache during the last year, 3793 (9.8%) fulfilled the criteria of migraine, 2139 (5.5%) had migraine with aura (MA), 1654 (4.3%) migraine without aura (MO), and 10,197 (26.3%) had other headaches.

### The association between elevated hs-CRP and headache

In the multivariate analyses, adjusting for age and sex, elevated hs-CRP was associated with any headache (OR 1.11, 95% CI 1.05–1.17), migraine (OR 1.23, 95% CI 1.13–1.34), MA (OR 1.26, 95% CI 1.13–1.40), MO (OR 1.17, 95% CI 1.03–1.32), and other headache (OR 1.08, 95% CI 1.02–1.14). In the final model 3, adjusting for all potential confounding factors, elevated hs-CRP was still significantly associated with migraine (OR 1.14, 95% CI 1.04–1.25) and MA (OR 1.25, 95% CI 1.03–1.29) (Table 2). In supplementary analyses, using individuals with “other headache “as reference category, elevated hs-CRP was more likely among those with MA (OR 1.17, 95% CI 1.05–1.31), but not MO (OR 1.04, 95% CI 0.92–1.19).

**Table 2:** Odds ratio (OR) with 95% confidence interval (CI) of high sensitivity C-reactive protein (hs-CRP) defined as > 3.0–20.0 mg/l related to headache types

	Total	High hs-CRP	Model 1	Model 2	Model 3
		N	OR (95% CI)	OR (95% CI)	OR (95% CI)
No headache	24,823	5179	1.00	1.00	1.00
Any headache	13,990	3029	1.11 (1.05–1.17)	1.07 (1.01–1.13)	1.05 (1.00–1.11)
< 1 days/month	3176	581	1.02 (0.95–1.11)	0.99 (0.91–1.07)	0.97 (0.90–1.08)
1–6 days/month	8099	1761	1.06 (0.99–1.13)	1.01 (0.95–1.08)	0.99 (0.93–1.07)
7–14 days/month	1731	425	1.28 (1.14–1.44)	1.21 (1.08–1.37)	1.15 (1.04–1.30)
≥ 15 days/month	984	262	1.40 (1.21–1.62)	1.32 (1.13–1.53)	1.26 (1.08–1.48)
P trend			< 0.001	0.001	0.01
Migraine	3793	864	1.23 (1.13–1.34)	1.17 (1.07–1.28)	1.14 (1.04–1.25)
< 1 days/month	599	115	0.99 (0.89–1.10)	0.94 (0.84–1.05)	0.92 (0.82–1.02)
1–6 days/month	2340	526	1.19 (1.07–1.31)	1.12 (1.01–1.25)	1.10 (1.00–1.23)
7–14 days/month	625	152	1.36 (1.12–1.64)	1.32 (1.09–1.60)	1.24 (1.02–1.51)
≥ 15 days/month	229	71	1.88 (1.42–2.50)	1.72 (1.29–2.31)	1.62 (1.21–2.17)
P trend			< 0.001	< 0.001	0.01
MA	2139	509	1.26 (1.13–1.40)	1.24 (1.11–1.39)	1.15 (1.03–1.29)
< 1 days/month	320	65	1.01 (0.90–1.13)	0.95 (0.85–1.07)	0.93 (0.82–1.04)
1–6 days/month	1290	293	1.19 (1.05–1.35)	1.13 (0.99–1.28)	1.09 (0.96–1.24)
7–14 days/month	391	105	1.56 (1.23–1.96)	1.51 (1.19–1.91)	1.40 (1.11–1.77)
≥ 15 days/month	138	46	2.15 (1.50–3.08)	2.01 (1.39–2.90)	1.84 (1.27–2.67)
P trend			< 0.001	< 0.001	0.002
MO	1654	355	1.17 (1.03–1.32)	1.12 (0.99–1.27)	1.09 (0.96–1.24)
< 1 days/month	279	50	0.99 (0.88–1.11)	0.94 (0.84–1.06)	0.92 (0.82–1.04)
1–6 days/month	1050	233	1.18 (1.03–1.35)	1.14 (0.99–1.31)	1.13 (0.98–1.30)
7–14 days/month	234	47	1.10 (0.79–1.52)	1.08 (0.77–1.50)	1.03 (0.74–1.44)
≥ 15 days/month	91	25	1.58 (0.99–2.51)	1.42 (0.88–2.28)	1.37 (0.85–2.22)
P trend			0.01	0.12	0.23
Other headaches	10,197	2165	1.08 (1.02–1.14)	1.04 (0.98–1.11)	1.04 (0.97–1.10)
< 1 days/month	2577	534	1.05 (0.96–1.14)	1.01 (0.93–1.10)	1.00 (0.92–1.09)
1–6 days/month	5759	1167	1.03 (0.96–1.11)	1.00 (0.93–1.07)	0.98 (0.91–1.05)
7–14 days/month	17,106	273	1.28 (1.11–1.47)	1.20 (1.04–1.39)	1.15 (1.00–1.33)
≥ 15 days/month	755	191	1.29 (1.09–1.53)	1.23 (1.04–1.46)	1.18 (0.99–1.41)
P trend			< 0.001	0.03	0.16

*MA* Migraine with aura, *MO* Migraine without aura. Model 1: Adjusted for ag, and sex. Model 2: Adjusted for age, sex and body mass index. Model 3: Adjusted for age, sex, body mass index, smoking, employment status, alcohol use, physical activity and Hospital Anxiety and Depression Scale score

No interaction was observed. As to the impact of headache frequency, the strongest association with elevated hs-CRP was found among individuals with headache ≥15 days/month for any headache (OR 1.26, 95% CI 1.08–1.48), migraine (OR 1.62, 95% CI 1.21–2.17), and MA (OR 1.84, 95% CI 1.27–2.67) (Table 2). A significant linear trend between increasing headache frequency and elevated hs-CRP was found for any headache (*p* = 0.01), migraine (*p* = 0.01) and MA (*p* = 0.002) (Table 2). No clear relationship was found between elevated hs-CRP and headache less than 7 days/month (Table 2).

### The influence of insomnia diagnosis

As demonstrated by Table 3, the relationship between elevated hs-CRP and headache was only found among subjects without insomnia, whereas no such association was found among those with insomnia. Supplementary analyses considering only those with headache 7–14 days/month and ≥ 15 days/month did not change the results (data not shown).

**Table 3:** Multi-adjusted^a^ odds ratio (OR) with 95% confidence interval (CI) of elevated high sensitivity C-reactive protein (hs-CRP) defined as > 3.0–20.0 mg/l related to headache types and insomnia status

	Total	Without insomnia		With insomnia	
		N	OR (95% CI)	N	OR (95% CI)
No headache	24,823	23,766	1.00	1057	1.00
Any headache	13,990	12,359	1.07 (1.01–1.13)	1631	0.83 (0.68–1.01)
Migraine	3793	3196	1.12 (1.02–1.24)	597	0.99 (0.76–1.20)
MA	2139	1777	1.19 (1.06–1.35)	362	0.97 (0.71–1.33)
MO	1654	1419	1.06 (0.92–1.22)	235	1.02 (0.72–1.45)
Other headaches	10,197	9163	1.06 (0.99–1.13)	1034	0.77 (0.62–0.96)

*MA* Migraine with aura, *MO* Migraine without aura ^a^Adjusted for age, sex, body mass index, smoking, employment status, alcohol use, physical activity and Hospital Anxiety and Depression Scale score

## Discussion

Cross-sectional population-based data based on a large number of participants showed that elevated hs-CRP was associated with headache ≥7 days/month, most evident for migraine with aura. No relationship was found between elevated hs-CRP and headache less than 7 days/month or with insomnia.

In agreement with the present results, a previous study have demonstrated that women with migraine had elevated CRP compared to women without migraine [4]. In a Dutch case-control study, individuals with MA were more likely to have elevated hs-CRP in the unadjusted analyses, and a borderline tendency was found in the multi-adjusted model [24]. In contrast, in a population-based study from Iceland, no difference in CRP levels were found between participants with and without migraine [6].

In the present study, the final multi-adjusted analyses showed that MA, but not MO, was associated with elevated hs-CRP. This is in contrast with a review of previous studies on this topic, where the association to MA was not found to be consistently stronger than to MO [26]. It should be highlighted that an association between hs-CRP and average number of aura attacks and total number of years with aura were reported in the Dutch study [24].

### Interpretation

In accordance with findings in the Tromsø 7 Study [29], MA was most consistently associated with elevated hs-CRP. It may be of relevance that hs-CRP is associated with risk of cardiovascular events [38], and that ischemic cardiovascular disease has a stronger association to MA than to MO [39].

In this large (*n* = 38,813) population-based study with presumably high statistical power, the association between elevated hs-CRP and headache was evident for those with headache 7–14 days/month and headache more than 14 days/month. Correspondingly, elevated hs-CRP was associated with migraine ≥7 days/month, but not migraine < 7 days/month in the Tromsø study (*n* = 20,486) [29]. This relationship with headache frequency and elevated hs-CRP has not been clearly demonstrated in other studies [3, 4, 6–8, 14, 18–22], possibly because of limited statistical power. The results of the present study may be explained by the finding that hs-CRP is related to general pain sensitivity [31]. Alternatively, our findings may be related to potential sterile inflammation in migraine [26] or other types of headaches, most prominent for those with chronic headache. However, whether sterile inflammation is an important pathological feature in migraine and other headaches is still debated.

We evaluated insomnia as a modifying factor on the association between hs-CRP and headache. In contrast to the results from the Tromsø 7 Study [26], no association was found between elevated hs-CRP and headache among those with insomnia. The reason for the contrasting finding is unclear. However, it may be a spurious finding in the Tromsø 7 study. An alternative explanation may be that there are differences between participants with insomnia in the Tromsø 7 study and in the HUNT3 study. It should be highlighted that the Tromsø Study was performed at almost 70 degree north, with midnight sun and winter darkness periods, whereas the HUNT3 study was performed in middle of Norway at 63–65 degrees north where the difference between summer and winter light is less extreme. The definition of insomnia in the Tromsø 7 study was stricter [29] than the one applied in the HUNT3 study [36], demanding symptoms three or more days per week instead of “several”, and with dissatisfaction with sleep quality as mandatory. Despite this, the prevalence of insomnia was higher in Tromsø 7 than in the HUNT3 study (8.1% vs. 6.9%, *p* < 0.001) [29, 36]. The prevalence reported in the present study is lower than other population-based studies in Norway [40], and we cannot exclude that our findings would have been different using another insomnia questionnaire.

The vast majority (93%) of the participants did not have insomnia, and in accordance with our main results, we found a relationship between elevated hs-CRP and headache also in this group.

### Strengths and limitations of the study

The present study shares several strengths and limitations with the study from Tromsø [29]. The major strengths of this study are the population-based design and the possibility to perform adjustments for all potential confounding factors previously included in the Tromsø study [29]. The large number of included participants give high statistical power and small confidence intervals.

The most important study limitation is the low participation rate (41%). Therefore, generalization of results should be performed with caution. It should also be highlighted that the cross-sectional design does not permit any conclusions about causality. Furthermore, it is a limitation that the diagnoses of headache and insomnia were questionnaire-based, which may have led to misclassification. On the other hand, the agreement between questionnaire-based diagnosis of migraine and interview has previously been found to be acceptable [33]. Insomnia was diagnosed by applying modified DSM-V criteria. DSM-V asks for symptoms three times or more per week [35], whereas the response option “several times per week” was used in the present study [36]. Furthermore, some types of daytime impairment are required in the DSM-V criteria [35]. In this respect it should be noted that our question “felt sleepy during the day?” may not have been optimal way to assess actual sleepiness, as it more probably reflects tiredness and fatigue [36].

## Conclusions

Cross-sectional data based on 38,007 participants showed that elevated hs-CRP was associated with headache ≥7 days/month, most evident for migraine with aura. Elevated hs-CRP was not associated with headache less than 7 days/month or with insomnia.

## Data Availability

Part of the dataset supporting the conclusions of this article is available on request to the corresponding author. Some of the data are the property of HUNT Research Centre and can only be accessed through direct contact with the research Centre.

## Abbreviations

BMI: Body mass index
CI: Confidence interval
DSM-V: Diagnostic and Statistical Manual of Mental Disorders, 5th ed
Hs-CRP: High sensitivity C-reactive protein
HUNT: The Nord-Trøndelag Health Survey
ICHD-2: The International Classification of Headache Disorders 2rd edition
MA: Migraine with aura
MO: Migraine without aura
OR: Odds ration
